# Editorial: Personalised multimodal prehabilitation in cancer

**DOI:** 10.3389/fonc.2022.1086739

**Published:** 2022-11-23

**Authors:** MA. West, F. Carli, M. P. W. Grocott

**Affiliations:** ^1^ School of Cancer Sciences, Faculty of Medicine, University of Southampton, Southampton, United Kingdom; ^2^ Perioperative and Critical Care Theme, National Institute for Health and Care Research (NIHR) Southampton Biomedical Research Centre, University Hospital Southampton/University of Southampton, Southampton, United Kingdom; ^3^ Department of Anesthesia, McGill University Health Center, Montreal, QC, Canada; ^4^ Integrative Physiology and Critical Illness Group, Clinical and Experimental Sciences, Faculty of Medicine, University of Southampton, Southampton, United Kingdom

**Keywords:** prehabiltiation, personalised, cancer, multimodal prehabilitation, multiphasic prehabilitation

Multimodality prehabilitation is a complex intervention that can enhance fitness, nutrition, and psychological resilience, with emerging evidence showing an improvement in perioperative and oncological outcomes (1). Personalised prehabilitation also has the potential to meet the widely adopted triple aim of health care: improving individuals’ experience of care, improving population health, and providing value for money to the taxpayer (2). The contemporary prehabilitation model has adopted a multimodal approach, which attempts to address complex needs in patients having complex treatment pathways. Multimodal prehabilitation incorporates intervention components specifically selected for their potential synergistic effects on health outcomes. Prehabilitation enables people with cancer prepare for treatment through promoting healthy behaviours and through needs-based prescribing of exercise, nutrition, and psychological interventions, aiming to empower patients to maximise resilience to treatment and improve long-term health outcomes (3). In this Research Topic entitled *‘Personalised Multimodal Prehabilitation in Cancer’* a collection of articles demonstrate how prehabilitation is now regarded as an integral part of a continuum spanning from cancer diagnosis to rehabilitation.

Multimodal prehabilitation interventions are often comprised of two or more of the following: i) aerobic and resistance training to attenuate cardiorespiratory and musculoskeletal deconditioning, ii) dietary interventions to counteract disease and/or treatment-related malnutrition, support anabolism and the metabolic cost of exercise; iii) psychological interventions to reduce stress, anxiety and associated morbidity; iv) the cessation of adverse health behaviours; and v) behavioural modification to support intervention initiation and adherence in the perioperative setting, whilst establishing self-management skills for long-term health behaviour change. In the perioperative setting, prehabilitation interventions are often combined with medical optimization of comorbidities (e.g., assessing/treating anaemia, diabetes and medication corrections) through collaboration with specialists expert in the management of long-term conditions

Santa Mina et al. set the scene by introducing the concept of multiphasic prehabilitation across the cancer continuum. Multiphasic prehabilitation is an innovative paradigm shift away from reactive assessments, that happen too late in the patient cancer pathway to be of any use in preparing or optimising patients for major surgery or cancer treatment, towards proactive early intervention. Multiphasic prehabilitation is intended to provoke investigation of proactive interventions that focus on periods of relative health where the ‘maximum tolerable dose’ for a health intervention can be pursued more readily in the absence of active treatments that often erode fitness, appetite, mental health and motivation (Santa Mina et al.). Multiphasic prehabilitation is individualised through screening and targeted assessment. This requires nuance and tailoring to the existing and anticipated experiences at each phase of the cancer journey to minimize treatment-related side effects and subsequent treatment delays, thereby improving wellbeing and potentially improving long term outcomes. The ‘aggressive’ push for patient preparation in this setting, may be akin to training models of high-performance sport with cyclic rounds of training prior to competition, both with similar goals: to optimize health preceding an anticipated stressor, ensuring ‘maximal performance’ and rapid recovery.

Waterland et al. illustrate the growing prehabilitation literature base and recent clinical recommendations in their updated systematic review and meta analyses. Prehabilitation improved preoperative functional capacity and substantially reduced hospital length of stay, however they did not find a significant reduction in postoperative complications, 30-day readmissions or postoperative mortality. They emphasise various points for future research including, the assessment of prehabilitation cost effectiveness, the need for new technology to tailor interventions and outcome, and measurement standardisation across the literature to allow for more efficient data utilisation and cleaner meta-analyses that minimises research waste. Mao et al. provide an interesting updated systematic review and meta-analysis illustrating that pulmonary rehabilitation is a meaningful addition to the whole perioperative patient pathway and demonstrating its value in reducing post-operative pulmonary complications. This review lends its support to previous literature in the pulmonary rehabilitation field, where exercise prescription has always been an integral component.

Brahmbhatt et al. demonstrated an improvement in functional capacity using a multimodal exercise intervention composed of resistance and aerobic exercise. Although not inherently novel, this interventional study carefully documents participant experiences interrogating intervention design preferences, perceived benefits, behaviour change, prehabilitation as education and the ability of patients to regain control. Unanimously, participants spoke positively about multimodality prehabilitation with inclusion of dietetic and psychological support to manage emotional stress and optimise health, echoing the concept of multiphasic prehabilitation across the cancer continuum (4). Importantly, prehabilitation was shown as a catalyst for positive healthy behaviour change, engagement, adherence and enabling patients to regain control of their disease process and importantly their cancer journey. Grimmett et al. describe the evidence on patient experiences and attitudes towards prehabilitation, with a specific focus on how behaviour science could strengthen uptake and adherence in prehabilitation programmes in both research and clinical settings. They also identify how behaviour change techniques (BCTs) represent ‘active ingredients’ in boosting the success of prehabilitation strategies, using goal setting, graded tasks and self-monitoring, to promote longer-term behaviour change. This can also be a research endpoint, where behavioural scientists may set out to understand behaviour strategies to improve motivation and compliance with the exercise component of prehabilitation. Working alongside clinical colleagues, behavioural scientists are well-placed to employ intervention mapping processes, behavioural analysis, and patient-centred intervention development. They can also provide training to colleagues delivering the programs to ensure the identified BCTs embedded within it are employed appropriately. Grimmett et al. highlight the importance of including qualitative process evaluation in the design of new prehabilitation trials as it together with BCTs, are vital to the integrity of the multimodal prehabilitation intervention and maximise positives outcomes.

Gillis et al. through their scoping review show that current prehabilitation literature lacks standardised and valid nutritional assessment methods, often coupled with interventions that lack an evidence basis. They conclude that nutrition interventions were inconsistently applied and lack adherence to accepted nutritional guidance published in oncology or surgery. Large gaps in the evidence exist in adopting validated nutritional screening and malnutrition assessment methods, interrogation of the ‘effect modification’ of a single interventional treatment effect on outcomes independent of nutritional status, intervention monitoring, adherence, evaluation, and cancer cohort heterogeneity. Gillis et al. go a step further in describing the nutrition care process for perioperative patients advocating its adoption in surgical prehabilitation. Here they emphasise the importance of developing a core outcome and measurement dataset to effectively address these substantial research gaps in surgical prehabilitation studies, allowing data meta-analyses and reduce research waste.

The implementation of prehabilitation in real-world clinical practice is limited, mainly due to logistical large-scale deployment, patient access and economical sustainability. These limitations can be overcome by innovative use of technology to facilitate screening, assessment, remote monitoring, and true personalisation of interventions. Barberan-Garcia et al. discuss the challenges in digital innovation in surgical prehabilitation, highlighting real-world issues and potential solutions e.g. digital physical activity prescription and behaviour change techniques aimed at improving perioperative outcomes. Digital technologies and remote monitoring is the future of true personalised prehabilitation.

We hope that this Research Topic presents a balanced view of the current challenges in cancer prehabilitation. We must move away from a ‘one size fits all’ approach and usher in a new era of individualised patient tailored multi-modal prehabilitation focussed on improving patient care and outcomes throughout the cancer journey.

## Author contributions

All authors listed have made a substantial, direct, and intellectual contribution to the work and approved it for publication.

## Conflict of interest

The authors declare that the research was conducted in the absence of any commercial or financial relationships that could be construed as a potential conflict of interest.

## Publisher’s note

All claims expressed in this article are solely those of the authors and do not necessarily represent those of their affiliated organizations, or those of the publisher, the editors and the reviewers. Any product that may be evaluated in this article, or claim that may be made by its manufacturer, is not guaranteed or endorsed by the publisher.
